# Tunning the Zeolitic Imidazole Framework (ZIF8) through the Wet Chemical Route for the Hydrogen Evolution Reaction

**DOI:** 10.3390/nano13101610

**Published:** 2023-05-11

**Authors:** Iqra Rabani, Supriya A. Patil, Muhammad Shoaib Tahir, Fatima Afzal, Je-Won Lee, Hyunsik Im, Young-Soo Seo, Nabeen K. Shrestha

**Affiliations:** 1 Department of Nanotechnology and Advanced Materials Engineering, Sejong University, Seoul 05006, Republic of Korea; iqrarabani@sju.ac.kr (I.R.); supriyaapatil11@gmail.com (S.A.P.); shoaibtahir10@gmail.com (M.S.T.); fati.physicist20@gmail.com (F.A.); wpdnjs97@sju.ac.kr (J.-W.L.); 2 Division of Physics and Semiconductor Science, Dongguk University, Seoul 04620, Republic of Korea; hyunsik7@dongguk.edu

**Keywords:** ZIF8, nanocrystals, HER, water splitting

## Abstract

Utilizing zeolitic imidazolate frameworks (ZIFs) poses a significant challenge that demands a facile synthesis method to produce uniform and nanometer-scale materials with high surface areas while achieving high yields. Herein, we demonstrate a facile and cost-effective strategy to systematically produce ZIF8 nanocrystals. Typically, ZIF8 nanocrystal synthesis involves a wet chemical route. As the reaction time decreased (150, 120, and 90 min), the size of the ZIF8 crystals decreased with uniform morphology, and productivity reached as high as 89%. The composition of the product was confirmed through XRD, FE-SEM, TEM, EDS, and Raman spectroscopy. The ZIF8 synthesized with different reaction time was finally employed for catalyzing the electrochemical hydrogen evaluation reaction (HER). The optimized ZIF8-3 obtained at 90 min of reaction time exhibited a superior catalytic action on the HER in alkaline medium, along with a remarkably long-term stability for 24 h compared with the other ZIF8 nanocrystals obtained at different reaction times. Specifically, the optimized ZIF8-3 sample revealed an HER overpotential of 172 mV and a Tafel slope of 104.15 mV·dec^−1^. This finding, thus, demonstrates ZIF8 as a promising electrocatalyst for the production of high-value-added green and sustainable hydrogen energy.

## 1. Introduction

The world’s energy resources, and fossil fuels are swiftly diminishing day by day. Additionally, climate concerns around the world also demand the mitigation of environmental pollution [1]. This issue has provoked researchers to ponder on alternative environmentally friendly energy resources [2]. Currently, the most promising is hydrogen owing to its exceptional caloric value, high energy density, cleanliness, and eco-friendliness [3,4,5,6,7,8,9,10,11,12,13,14]. Although there are many techniques for hydrogen production, the electrochemical hydrogen evolution reaction (HER) from water splitting is an eco-friendly and zero-emission technology. However, this approach requires costly noble (Pt and Ru) metals and metal-based compounds for successful electrocatalysis [15,16,17,18]. These noble metals are rare and valuable; moreover, they deteriorate during electrochemical operations. As a result, some low-cost alternatives with high catalytic activity and long-term durability are required to limit the usage of noble metals. Although metal-based catalysts such as sulfides, phosphides, transition metal nitrides, and metal-free catalysts remain significant in the HER, their catalytic activity makes them inferior to Pt-based catalysts [19,20,21,22].

Metal–organic frameworks (MOFs) are a class of porous materials composed of metal ions or clusters connected by organic ligands [23]. They have unique properties that make them attractive for a range of applications, including catalysis, gas storage, separation, and sensing [24,25,26]. Interestingly, MOFs have flexible structures, variable chemical composition, high surface area, an affinity for specific substances, and functional pore sidewalls. During the last decade, MOF-based hybrid materials, such as zeolite imidazole frameworks (ZIFs), have been extensively investigated due to their special morphology and excellent stability in water and wastewater treatment [27]. In addition, ZIF8 has a distinctive porous structure, with particular selectivity and ample active sites for adsorption [28]. Furthermore, the ZIF8 crystal structure offers good thermal and chemical stability owing to the existence of metal–imidazole bonds and hydrophobic porous walls [27]. In order to fulfill the catalytic activity for HER, various attempts have been made to incorporate MOFs with composite materials having active metal species [29].

Although substantial studies have been published on the morphological features of ZIF8, only a few have been reported on the consequences of morphology for the HER. Chen and coworkers adopted a hydrothermal process along with an in-situ ion-exchange mechanism to prepare ZnO@ZIF8 nanorod arrays possessing catalytic activity for the HER in acid medium [29]. Sun et al. synthesized an Rh-based ZIF8 catalyst and recorded the HER efficiency in 0.5 M H_2_SO_4_ electrolyte; it proved to be a promising catalyst by showing a superior HER rate [30].

In this study, a simple, green, and cost-effective route for the fabrication of hexagonal ZIF8 nanocrystals is demonstrated systematically. As an efficient electrocatalyst for the HER, the synthesis conditions were optimized by altering the reaction kinetics via the reaction time. Interestingly, the optimized ZIF8-3 with a reaction time of 90 min had the smallest crystal size with uniform morphology. The optimized ZIF8-3 crystals demonstrated a low HER overpotential of 170 mV and a low Tafel slope of 104.15 mV·dec^−1^, with long-term electrochemical durability.

## 2. Materials and Methods

### 2.1. Materials

All chemicals utilized in this study were commercially purchased from suppliers and employed without further purification. 2-Methylimidazole (2-MIM, 99%), zinc nitrate hexahydrate (Zn(NO_3_)_2_·6H_2_O, 99%), and methanol (MeOH, 99.99%) were purchased from Sigma Aldrich, South Korea. Prior to employing it as the current collector substrate, nickel foam (NF, 1.6 mm thickness) obtained from ALANTUM, South Korea, was carefully cleaned with HCl, ethanol, acetone, and deionized (DI) water.

### 2.2. Synthesis of ZIF8 Crystals

**Scheme 1:**
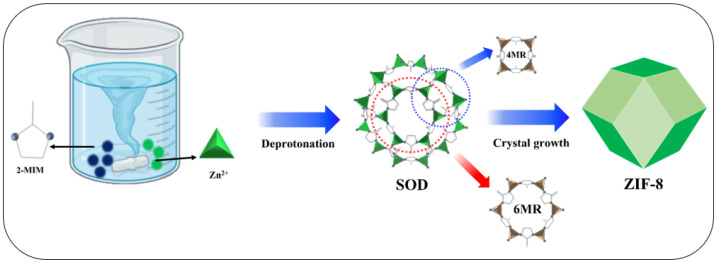
Schematic illustration of the formation of sodalite structured ZIF8.

The wet chemical method was used to synthesize ZIF8 with controlled morphology by varying the reaction time, and the samples were named ZIF8-1–3. Scheme 1 illustrates the synthesis process of ZIF8. In this method, 2-methylimidazole was dissolved in MeOH, and Zn salt was separately mixed in MeOH. The former solution was then abruptly poured into the latter solution with varying reaction times of 150, 120, and 90 min at a continuous stirring speed of 350 rpm. The resulting white precipitates were collected and washed thrice with MeOH. The final product, ZIF8 crystals, was collected by drying in a vacuum oven overnight at 75 °C. The complete experimental details are presented in Table 1.

### 2.3. ZIF8 Characterization

The morphology of the as-prepared samples was investigated using field-emission scanning electron microscopy (FE-SEM; Hitachi (Tokyo, Japan), SU-8010, 5.0 kV, working distance: 6.8 mm) and high-resolution transmission electron microscopy (TEM; JEM-2010, 200 kV). Chemical composition analysis was performed using the energy dispersive X-ray (EDX) technique operating at 5 kV and 10 keV. The vibrational modes were determined in the range between 200 and 1200 cm^−1^ via a Renishaw Raman spectrometer (RM 200, England). The surface chemical composition and valence-states of the diverse elements were investigated by means of X-ray photoelectron spectroscopy (ESCALAB Xi, Thermo-Fischer Scientific, Waltham, MA, USA) at 1486 eV of Al Kα energy as signal excitation source. X-ray diffraction spectroscopy (Panalytical XRD-6100 instrument with Cu Kα radiation (λ = 1.5406 Å) at a scan speed of 5°/min; Panalytical, Malvern, UK) was used for the investigation of the crystal structure. The surface characteristics of the as-prepared samples were studied by nitrogen (N_2_) adsorption/desorption isotherms (BET, Micrometrics-ASAP-2020; Micromeritics Instrument, Norcross, GA, USA).

### 2.4. Electrochemical Measurement

All required HER characteristics were analyzed using an electrochemical workstation (BioLogic potentiostat). The catalytic HER half-reactions of water splitting were analyzed at room temperature using a three-electrode system consisting of the ZIF8 deposited on an NF substrate as a working electrode, graphite rod as a counter electrode, and saturated calomel electrode (SCE) as a reference electrode. All electrochemical measurements were performed at a scan rate of 5 mV·s^−1^ in an alkaline 1.0 M KOH electrolyte. The active material (electrocatalysts) was mixed with carbon black and polyvinylidene fluoride (PVDF) in a ratio of 80:10:10 in a mortar with the help of a few drops of N-methyl-2-pyrrolidone (NMP); the slurry was decorated on a NF substrate using a makeup brush, and finally dried in an oven overnight at 100 °C. The catalytic electrodes were activated via cyclic voltammetry (CV) at 100 mV·s^−1^ until stable linear sweep voltammetry (LSV) curves were obtained.

To prepare the benchmark Pt electrode, a homogeneous catalyst ink was prepared by dispersing 10 mg of Pt/C in 1.80 mL of ethanol mixed with 20 µL of Nafion (5 wt.%) for 1 h using a sonicator. Furthermore, to achieve the loading quantity of 1.0 mg·cm^2^, the produced ink (200 µL) was drop-casted into the surface of a NF (1 × 1 cm^2^). The electrode potential was converted to the reversible hydrogen electrode (RHE) as follows:

E_RHE_ = E_SCE_ + E°_SCE_ + (0.0591) pH, (1)

where E°_SCE_ is taken as 0.241 V.

Electrochemical impedance spectroscopy (EIS) was performed in the same electrochemical working station and recorded between 1 and 10 k Hz with 10 mV of an applied AC signal amplitude. The linear sweep voltammetry (LSV) data were iR-corrected by subtracting the ohmic resistance losses. To evaluate the electrochemical active surface area (ECSA), the electrochemical double-layer capacitance (C_dl_) was acquired using CV in a non-Faradaic region at 10, 20, 30, 40, and 50 mV·s^−1^. The C_dl_ values were determined from the slope of a straight line obtained by plotting Δj {(ja − jc)/2} vs. scan rate, where ja and jc represent the anodic and cathodic current densities.

## 3. Results

### 3.1. Morphology and Structural Analysis

**Figure 1:**
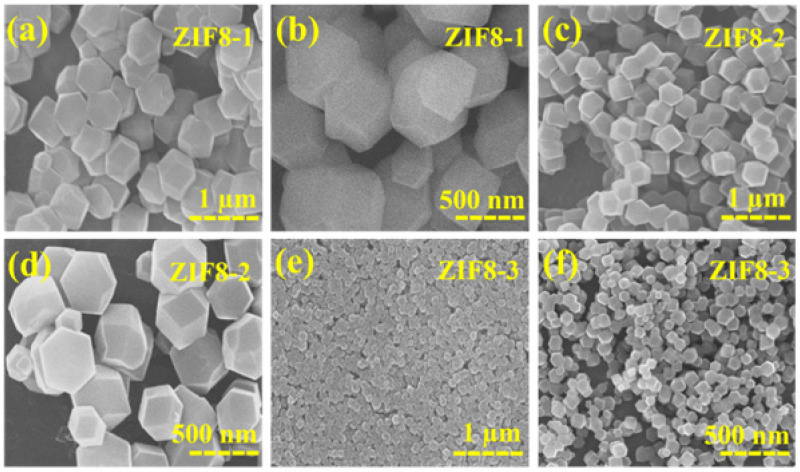
Field-emission electron microscopy (FE-SEM) images at high and low magnifications of (**a**,**b**) ZIF8-1 (150 min), (**c**,**d**) ZIF8-2 (120 min), and (**e**,**f**) ZIF8-3 (90 min).

In this study, FE-SEM analysis was conducted to investigate the surface morphology of synthesized ZIF8. Figure 1a–f shows the morphology of ZIF8 nanocrystals. Regardless of the synthesis time, highly crystalline ZIF8 crystals were observed with a well-defined hexagonal shape. To optimize the hexagonal crystal growth for the electrocatalysis purpose, the reaction time was varied from 150 to 90 min. Upon shortening the reaction time, no well-defined solid precipitate was obtained when the reaction was performed for less than 90 min. Hence, no further experiments were performed for the synthesis and characterization of samples at a reaction time shorter than 90 min. The maximum size of ZIF8-1 crystals was observed at a reaction time of 150 min with a crystal size of approximately 750 nm, as presented in Figure 1a,b. The reaction time was then shortened to 120 min (Figure 1c,d), resulting in the growth of smaller ZIF8-2 crystals with a crystal size of approximately 630 nm. Micrographs of ZIF8-3 obtained at 90 min are shown in Figure 1e,f, revealing a further reduction in crystal size to 70 nm. Notably, the ZIF8-3 crystals still maintained a highly crystalline well-defined hexagonal morphology despite their smaller size. The decrease in crystal size is attributed to the high nucleation rate of ZIF8 crystals at the beginning of the synthesis reaction, resulting in the formation of many small crystals that merge to form a large crystal as the reaction progresses [31]. XRD analysis correlated well with the FE-SEM results, and the average crystal size was found to be ~750 nm, ~630 nm, and ~70 nm, for ZIF8-1, ZIF8-2, and ZIF8-3, respectively.

**Figure 2:**
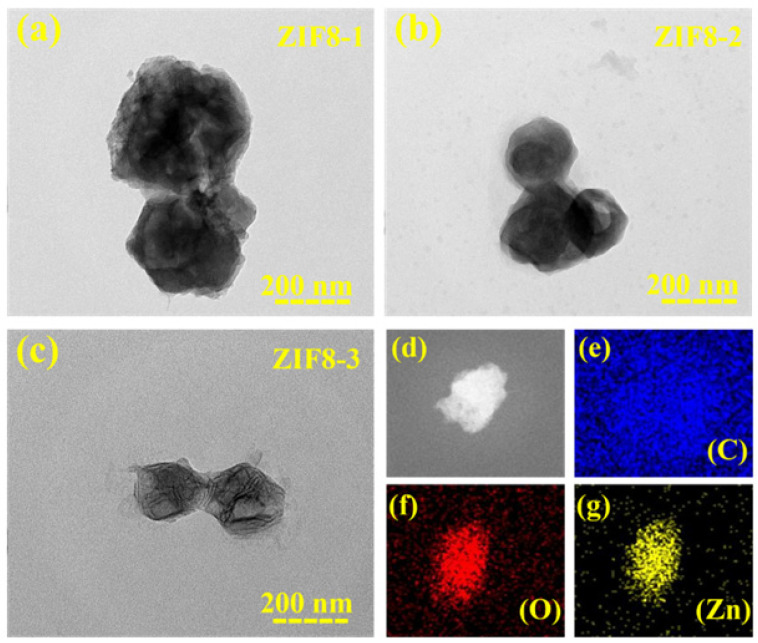
Transmission electron microscopy (TEM) micrographs of (**a**) ZIF8-1 (150 min), (**b**) ZIF8-2 (120 min), and (**c**) ZIF8-3 (90 min); (**d**–**g**) EDS mapping of optimized ZIF8-3 nanocrystals (Bar scale: graph 200 nm).

Furthermore, the surface morphology of the synthesized ZIF8 nanocrystals was analyzed using transmission electron microscopy (TEM). Figure 2a–d present the TEM micrographs of the ZIF8 nanocrystals at various magnifications.

The results showed that ZIF8-1 nanocrystals had a hexagonal shape. The crystal morphology and the sizes obtained at different reaction times were in good agreement with the SEM results. Although the hexagonal morphology was maintained, in addition to the crystal size, a slight deterioration of sodalite morphology upon shortening the reaction time from 150 to 90 min could be observed. Moreover, the crystallinity of the crystals was also slightly reduced due to the initial nucleation [32,33], as observed in Figure 2c. To further see the spatial distribution of the ZIF8-3, the elemental mapping EDX was performed at the specific area of the TEM micrograph, as shown in Figure 2d. Figure 2e–g shows the EDS mapping of the optimized ZIF8-3 nanocrystals, demonstrating the elemental distribution of C, O, and Zn. The TEM and EDS images confirmed the successful formation of the required ZIF8 nanostructures.

**Figure 3:**
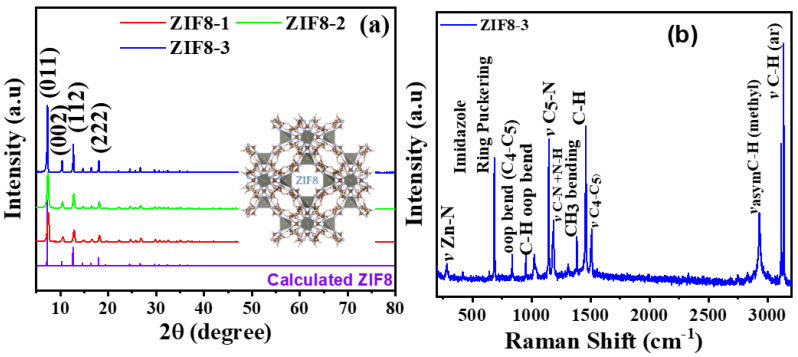
(**a**) X-ray diffraction patterns of ZIF8-1, ZIF8-2, and ZIF8-3. The inset figure is a 3D-SOD topology view of ZIF8 along crystallographic a-axis 8. (**b**) High-resolution Raman spectrum for optimized ZIF8-3.

The crystal structure of the as-synthesized ZIF8 nanocrystals under various synthesis conditions was confirmed by X-ray diffraction (XRD), and the corresponding results are shown in Figure 3a. The prominent reflection peaks at 7.21°, 10.42°, 12.81°, 14.57°, and 17.8° corresponding to ZIF8 were observed with crystal planes of (011), (002), (112), and (222) [34,35]. The presence of the (002) reflection peak in all samples confirmed the correct formation of ZIF8 nanocrystals [36]. The hexagonal structure of ZIF8 crystal was cross-verified with JCPDS card no. 00-062-1030 and compared with the calculated ZIF8 pattern obtained from the reported CIF file (CCDC 899389) [37,38,39]. As evident in Figure 3a, all XRD patterns obtained experimentally matched well with the calculated XRD patterns. However, gradual sharp peaks with low central width were observed moving from ZIF8-1 to ZIF8-3 due to the reduced crystal size, indicating a decrease in crystallinity of ZIF8 nanocrystals upon shortening the reaction time. ZIF8 crystallized in a cubic crystal system with the I-43m space group symmetry having the topology shown in the inset of Figure 3a.

In addition to the crystal structure, the surface chemistry of the optimized ZIF8-3 crystals was analyzed by Raman spectroscopy. The high-resolution Raman spectrum (Figure 3b) with an incident laser of 633 nm exhibited the number of peaks corresponding to stretching, bending, out-of-plan, aromatic, and asymmetric bonds of the ZIF8-3 molecules [40]. The band assignments are provided in Supplementary Table S1 in detail according to the previously published literature [40,41]. The band at 282 cm^−1^ was attributed to Zn–N stretching, while the bands observed at 686 cm^−1^, 1146 cm^−1^, and 1460 cm^−1^ were assigned to imidazolium ring puckering, C5–N stretching, and methyl bending, respectively. The antisymmetric stretching band in C–H by the methyl group was found at 2930 cm^−1^, and the C–H stretching vibration band of the imidazole ring was found at 3131 cm^−1^. A slight shift in peaks was observed due to the different crystal sizes [35,42]. The availability of all these bands verified the correct formation of ZIF8-3.

**Figure 4:**
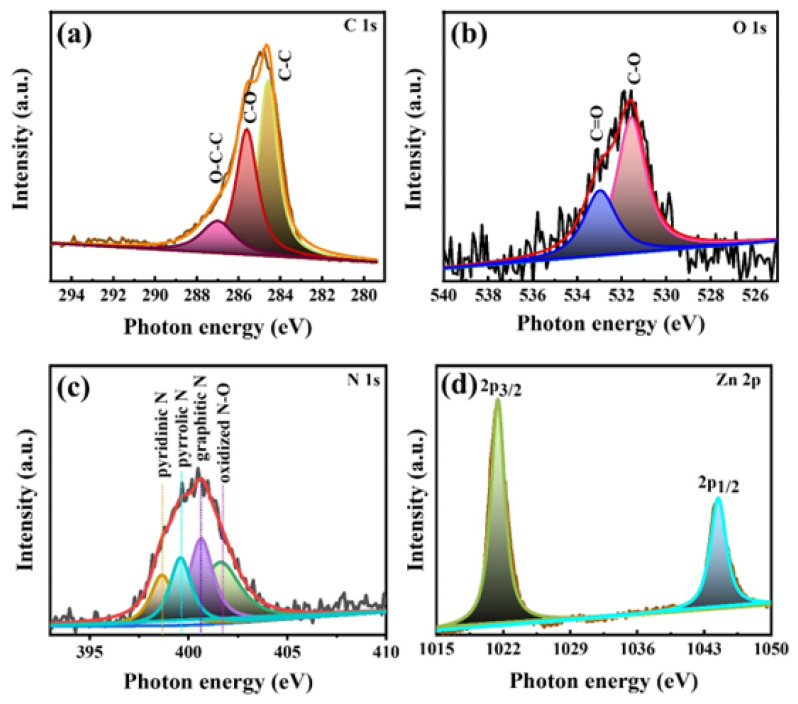
High-resolution XPS profiles for the optimized ZIF8-3 nanocrystals: (**a**) C 1*s* region, (**b**) O 1*s* region, (**c**) N 1*s* region, and (**d**) Zn 2p region.

The chemical composition and valence electronic states of the optimized ZIF8-3 nanocrystal were evaluated using high-resolution X-ray photoelectron spectroscopy (XPS), and the results are illustrated in Figure 4a–d. Supplementary Information (Figure S1) provides the detailed XPS survey scan, revealing the existence of C, O, N, and Zn elements in ZIF8. The high-resolution C 1*s* spectrum revealed peaks at 284.5, 285.6, and 287.1 eV corresponding to C–C, C–O, and O–C–C binding, respectively, as shown in Figure 4a [25]. As evident in the high-resolution O 1*s* spectrum shown in Figure 4b, the peaks obtained at 531.5 and 533.0 eV binding energies were associated with C–O and C=O, respectively [25]. The high-resolution N 1*s* spectrum (Figure 4c) revealed four peaks of pyridinic N (397.4 eV), pyrrolic N (398.0 eV), graphitic N (399.1 eV), and oxidized N (400.1 eV) [26]. Figure 4d further presents the Zn 2p high-resolution XPS profile, with peaks at binding energies of 1044.5 eV and 1021.5 eV, associated with the Zn 2p_1/2_ and Zn 2p_3/2_ states, respectively. Therefore, the spin–orbit splitting of Zn 2p (2p_1/2_ and 2p_3/2_) was 23 eV, demonstrating the oxidation state of Zn^2+^ [26]. This finding reveals that the ZIF8-3 nanocrystals were successfully synthesized through the chemical wet method.

**Figure 5:**
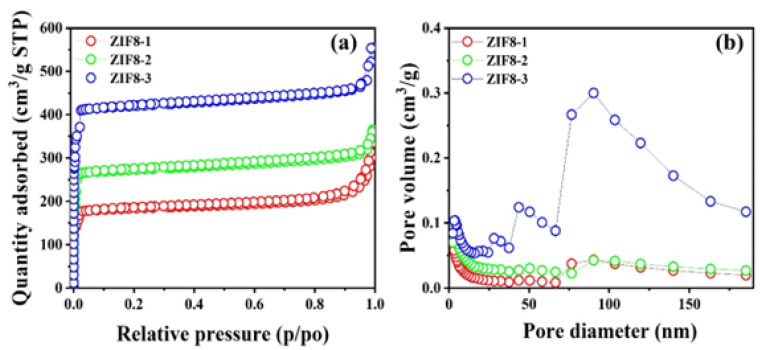
Surface analysis of the as-synthesized ZIF8 nanocrystals: (**a**) N_2_ adsorption/desorption isotherms for ZIF8-1, ZIF8-2, and ZIF8-3; (**b**) BJH curves for ZIF8-1, ZIF8-2, and ZIF8-3.

The specific surface area with related physical parameters was assessed using BET analysis. The N_2_ adsorption–desorption isotherms of the as-synthesized ZIF8 nanocrystals (ZIF8-1, ZIF8-2, and ZIF8-3,) showed type IV hysteresis [43,44], indicating the mesoporous nature of the materials according to the IUPAC classifications; the results are illustrated in Figure 5a. The cumulative pore volume and pore size distributions were analyzed from the Barrett–Joyner–Halenda (BJH) profiles (Figure 5b). Thus, the respective specific surface areas of the as-synthesized ZIF8-1, ZIF8-2, and ZIF8-3 were 1166, 1233, and 2100 m^2^/g, with corresponding pore volumes of 0.241, 0.623, and 1.73, cm^3^/g, respectively. The surface area and pore volume of the optimized ZIF8-3 were higher than those of ZIF8-1, and ZIF8-2. The higher specific surface area of ZIF8-3 provided a larger electrolyte/electrode contact area, resulting in accessibility to more active sites and suggesting excellent HER performance [45].

### 3.2. HER Performance of the As-Synthesized ZIF8

**Figure 6:**
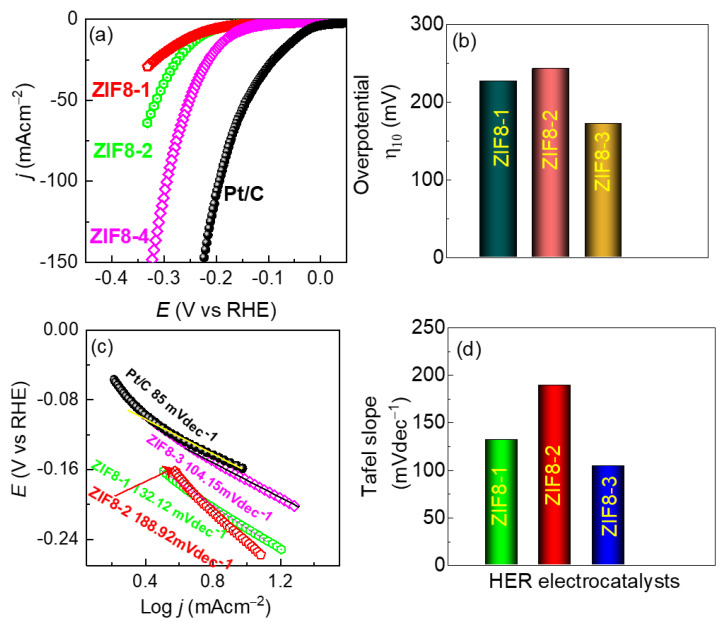
(**a**) HER polarization curves of 90 min, 120 min, and 150 min reaction time-based catalysts, and Pt/C at a scan rate of 5 mV·s^−1^ in 1.0 M KOH. (**b**) Comparative overpotential vs. fabricated catalyst at a current density of 10 mA·cm^−2^. (**c**) Corresponding Tafel slope extracted from HER LSV curve. (**d**) Tafel slope values vs. HER electrocatalyst.

To investigate the electrocatalytic performance of the ZIF8 samples in promoting hydrogen evolution, the samples were subjected to cathodic polarization in an alkaline 1.0 M KOH electrolyte against a standard calomel electrode (SCE) and a graphite counter electrode. The obtained LSV curves are shown in Figure 6a. The LSV polarization curve demonstrated that the ZIF8-3 sample-based cathode showed higher electrocatalytic activity than the ZIF8-1 and ZIF8-2 catalyst-based cathodes. Interestingly, to drive the current density of 10 mA·cm^−2^, the ZIF8-1, ZIF8-2, and ZIF8-3 sample-based cathodes showed an HER overpotential of 227, 243, and 172 mV, respectively. Remarkably, the overpotential of the ZIF8-3 electrode was lower than that of other catalysts, revealing that the 90 min reaction time-based catalyst (ZIF8-3) offered enhanced HER electrocatalytic activity. Figure 6b compares the details of HER catalytic activity in terms of the overpotential of all prepared catalysts at 10 mA·cm^−2^.

Moreover, the Tafel slopes of the catalysts were applied to judge the reaction HER kinetics and the mechanism involved. The HER mechanism is commonly recognized to occur on the cathode surface via a multistep electrochemical reaction process. To be more specific, the multistep HER electrochemical process in an alkaline electrolyte medium involves the following reaction stages:

(2) $$H_{2}O+M+e^{-}\to {\mathrm{MH}}_{\mathrm{ads}}+{\mathrm{OH}}^{-}\;\left(\mathrm{Volmer}\right),$$

(3) $${\mathrm{MH}}_{\mathrm{ads}}+H_{2}O+e^{-}\to M+{\mathrm{OH}}^{-}+H_{2}\;\left(\mathrm{Heyrovsky}\right),$$

(4) $${\mathrm{MH}}_{\mathrm{ads}}\to 2{\mathrm{MH}}_{2\;}\left(\mathrm{Tafel}\right),$$

where “M” refers to a vacant electrocatalyst surface site, while “MH_ads_” refers to the absorbed hydrogen atoms. The hydrogen evolution reaction (HER) mechanism on ZIF8-3 involves the transfer of electrons to protons to produce hydrogen gas. The active sites of ZIF8, which are normally the metal ions coordinated to the imidazole ligands, are exposed to proton adsorption from the electrolyte solution. The active sites on ZIF8 receive electrons from the electrode, which are then used to reduce the adsorbed protons to hydrogen atoms. The imidazole ligands act as electron donors, making it easier for electrons to be donated to the hydrogen atoms that are adsorbed. Adsorbed hydrogen atoms spread around the catalyst’s surface in search of an appropriate location for combination. A hydrogen molecule (H_2_) is created when two adsorbed hydrogen atoms combine and are subsequently liberated from the catalyst surface. The HER mechanism on ZIF8 generally entails the adsorption and activation of protons on the metal ions coordinated to the imidazole ligands, followed by the transfer of electrons to decrease the deposited protons to hydrogen atoms. After diffusing and combining, the hydrogen atoms produce hydrogen molecules, which are subsequently released from the catalyst’s surface. ZIF8-3′s strong catalytic activity for the HER reaction results from its distinctive structural and electrical characteristics, including its porous structure and metal–ligand coordination [45]. Figure 6c shows the Tafel plots of all fabricated samples. The ZIF8-3 sample showed a Tafel slope of 104.15 mV·dec^−1^, which was much lower than that of the ZIF8-1 (132.12 mV·dec^−1^) and ZIF8-2 (188.92 mV·dec^−1^), samples, but higher than the Pt/C (85 mV·dec^−1^) benchmark electrode. The smaller Tafel slope of the ZIF8-3 sample was attributable to the improved electrochemical HER kinetics, linked to the increase in electrocatalytically active sites of the catalyst sample. Figure 6d shows a detailed profile of the Tafel slope for the ZIF8 catalysts synthesized in different conditions.

The electrochemical active surface areas (ECSAs) were used to calculate the associated active sites of the electrodes determined by the double-layer capacitance (C_dl_), which was derived from the non-faradaic CV curve region for each electrode in the 1.0 M KOH electrolyte with various scan speeds as shown in Supplementary Figure S2. ECSA was determined from Equation (5) [46,47].

(5) $$\mathrm{ECSA}=\frac{C_{\mathrm{dl}}}{C_{s}},$$

where C_s_ is the specific capacitance of the electrode, typically reported as 0.040 mF·cm^−2^ in 1.0 M KOH electrolyte [47].

**Figure 7:**
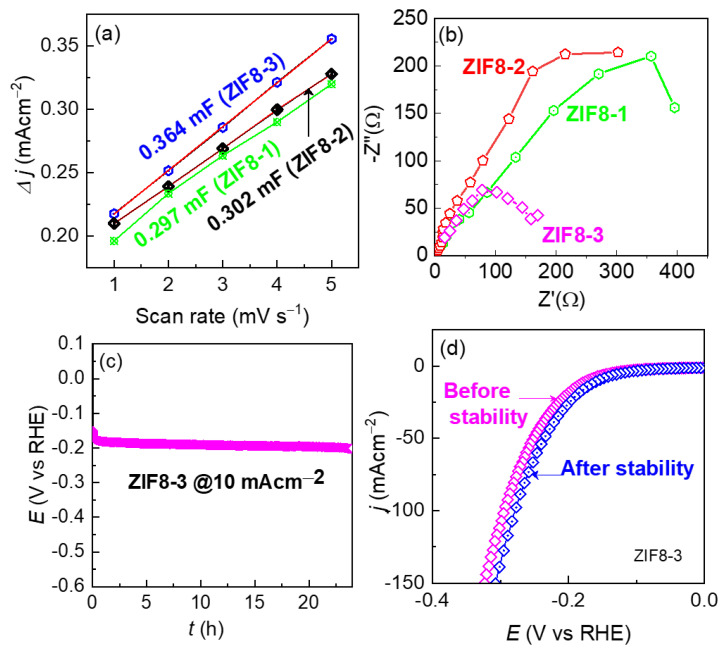
(**a**) Linear plots illustrating the extraction of the double-layer capacitance (C_dl_) for each ZIF8 electrode. (**b**) Nyquist plots of ZIF8 electrode. (**c**) Chrono potentiometric measurement curve for the ZIF8-3 (90 min) electrode measured in alkaline medium at 10 mA·cm^−2^ current density. (**d**) Polarization curves for the ZIF8-3 electrode before and after the stability test in alkaline medium.

According to Figure 7a, the ZIF8-3 electrode exhibited a larger ECSA (9.123 cm^−2^) than the ZIF8-2 (7.55 cm^−2^) and ZIF8-1 (7.425 cm^−2^) samples. Furthermore, to investigate the charge-transfer (R_ct_) resistance between the catalyst electrode and the electrolyte, electrochemical impedance spectroscopy (EIS) was employed. Figure 7b shows that the ZIF8-3 sample had the smallest semicircle arc on the Nyquist plot, revealing that it exhibited the smallest Rct resistance for the HER. Specifically, the ZIF8-3 sample showed a charge transfer resistance of 61.09 Ω, which was significantly lower than that for the ZIF8-1 (84.98 Ω) and ZIF8-2 (74.89 Ω) samples, respectively.

Another crucial factor in assessing the applicability of a suitable catalyst is the long-term electrochemical stability. As evident in Figure 7c, the ZIF8-3 catalyst-based cathode displayed a strong chronopotentiometry HER stability for the continuous electrolysis time set for 24 h at 10 mA·cm^−2^. In addition, the LSV curves (Figure 7d) of the ZIF8-3 sample before and after the stability test were very close to each other. This further justifies the sustainability of the ZIF8-3 catalyst for the HER.

On the basis of the results presented above, it can be concluded that a facile reaction kinetic tunning via reaction time variation for the growth of ZIF8 crystals through a wet chemical synthesis can be an effective approach for scrutinizing the electrocatalytic activity of the ZIF8. These findings could offer new opportunities for the development of nonprecious MOF materials electrocatalysts for green energy conversion.

To examine the impact of prolonged HER operation, we analyzed the ZIF8-3/NF anode using SEM (refer to Supplementary Figure S3). Even after the long-term stability test, we could observe the crystal structure of ZIF8-3 from the bulk phase of the catalyst. However, the SEM surface image of the electrode (shown in Supplementary Figure S3a,b) indicates that the hexagonal shape morphology of the electrode partially collapsed.

## 4. Conclusions

In conclusion, a facile strategy for the synthesis of ZIF8 nanocrystals with excellent water-splitting outcomes via the HER was designed with a simple and green wet chemical approach. The crystal size of the ZIF8 and, hence, the HER catalytic activity was tuned simply by varying the reaction time. When the reaction time was varied from 150 min to 90 min, the crystal size was reduced with a slight deterioration of sodalite morphology. The deterioration morphology of these nanocrystals was due to the quick nucleation with the access of an imidazole linker. The optimized ZIF8-3 (90 min reaction time) electrode showed an enhanced electrocatalytic HER response with a minimal overpotential of 172 mV at a driving current of 10 mA·cm^−2^. In addition, the ZIF8-3 catalyst showed a low Tafel slope of 104.15 mV·dec^−1^. Lastly, the optimized ZIF8-3 catalyst demonstrated enormous electrocatalytic HER stability up to 24 h at a current density of 10 mA·cm^−2^.

## Figures and Tables

**Table 1:** Experimental conditions and crystal size variation of synthesized ZIF8. The optimized sample is highlighted with bold.

Experiments	Sample	2-MIM:Zn (NO_3_)_2_·6H_2_O	Crystal Size (nm)	Reaction Time (min)
1	ZIF8-1	1.10:1	~700–800	150
2	ZIF8-2	1.10:1	~400–550	120
**3**	**ZIF8-3**	**1.10:1**	**~70–100**	**90**

## Data Availability

The data presented in this study are available in this article and Supplementary Materials.

## Supplementary Materials

The following supporting information can be downloaded at: https://www.mdpi.com/article/10.3390/nano13101610/s1, Table S1: Raman Band Assignments of ZIF8-3; Figure S1: Survey XPS spectra for the optimized ZIF8-3; Figure S2: Cyclic voltammetry (CV) curves of (a) ZIF8-3, (b) ZIF8-1, and (c) ZIF8-2, electrodes materials recorded at different scan rates of 10–50 mVs^−1^; Figure S3: (a,b) Post-stability SEM images of ZIF8-3 at different magnifications.
