# Supplementary material for: Tunning the Zeolitic Imidazole Framework (ZIF8) through the Wet Chemical Route for the Hydrogen Evolution Reaction
Source: Nanomaterials (Basel). 2023 May 11;13(10):1610. doi: 10.3390/nano13101610 (PMC10222776; doi:10.3390/nano13101610)
Supplement: Supplementary file 1 [file nanomaterials-13-01610-s001.zip › nanomaterials-2381291-supplementary.pdf]

Supporting Information

Tunning the Zeolitic Imidazole Framework (ZIF8) through the Wet Chemical Route for the Hydrogen Evolution Reaction

Iqra Rabani [1,†], Supriya A. Patil [1,†], Muhammad Shoaib Tahir [1], Fatima Afzal [1], Je-Won Lee [1], Hyunsik Im [2], Young-Soo Seo [1,*] and Nabeen K. Shrestha [2,*]

[1] Department of Nanotechnology and Advanced Materials Engineering, Sejong University, Seoul 05006, Republic of Korea; iqrarabani@sju.ac.kr (I.R.); supriyaapatil11@gmail.com (S.A.P.); shoaibtahir10@gmail.com (M.S.T.); fati.physicist20@gmail.com (F.A.); wpdnjs97@sju.ac.kr (J.-W.L.)

[2] Division of Physics and Semiconductor Science, Dongguk University, Seoul 04620, Republic of Korea; hyunsik7@dongguk.edu

* Correspondence: ysseo@sejong.ac.kr (Y.-S.S.); nabeenkshrestha@dongguk.edu (N.K.S.)

† These author contributed equally to this work.

Table S1. Raman Band Assignments of ZIF8-3.

Frequency (cm^{-1})	Band Assignments [a]
273	ν Zn−N
686	Imidazole ring puckering, H oop bend
833	C−H oop bend (C4−C5)
1021	C−H oop bend
1146	ν C5−N
1187	ν C−N+N−H wag
1311	ring expansion + N−H wag
1385	CH3 bending
1460	C-H wag
1508	ν C4−C5
2930	ν_{asym} C−H (methyl)
3131	ν C−H (ar)

[a] ν: stretching, oop: out of plane, ar: aromatic, asym: aymmetric.

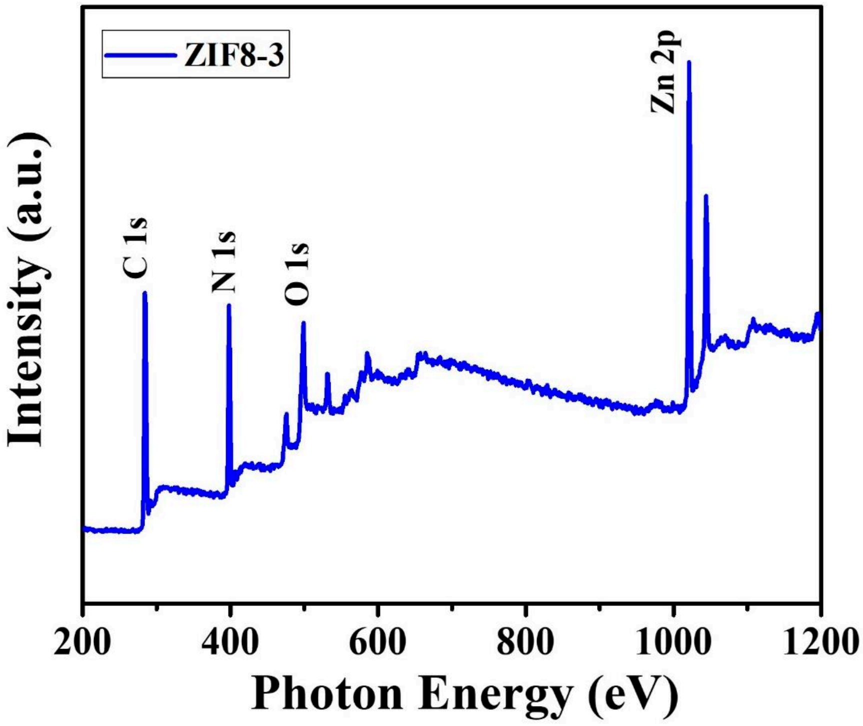

Figure S1. Survey XPS spectra for the optimized ZIF8-3.

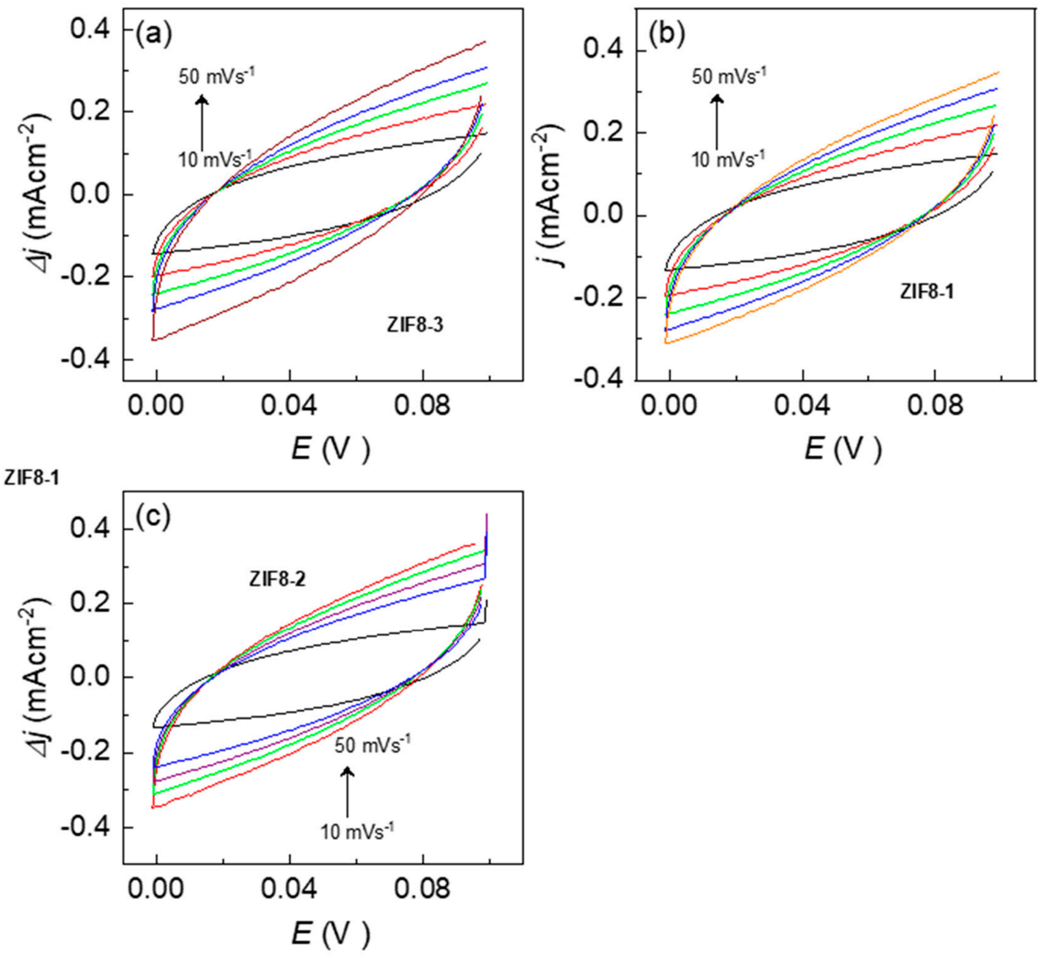

Figure S2. Cyclic voltammetry (CV) curves of (**a**) ZIF8-3, (**b**) ZIF8-1, and (**c**) ZIF8-2, electrodes materials recorded at different scan rates of 10-50 mVs^{-1}.

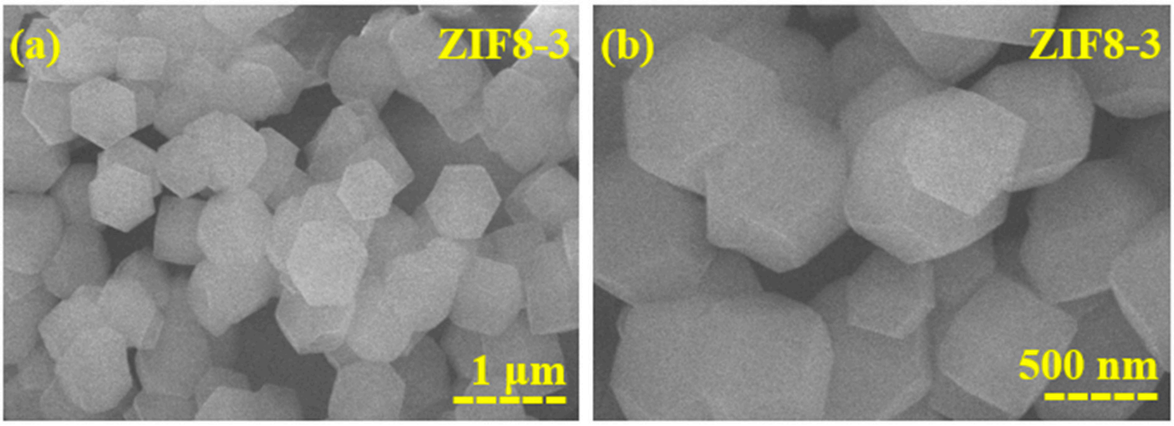

Figure S3. (**a**,**b**) Post-stability SEM images of ZIF8-3 at different magnifications.
